# Acute mesenteric ischemia following lancehead snakebite: an unusual case report in the Northernmost Brazilian Amazon

**DOI:** 10.3389/fmed.2023.1197446

**Published:** 2023-06-22

**Authors:** Luis E. B. Galan, Vitória Souza Silva, Vitória Santos Silva, Rommel C. Monte, Sewbert R. Jati, Isadora S. Oliveira, Felipe A. Cerni, Wuelton M. Monteiro, Jacqueline Sachett, Domingos S. M. Dantas, Roberto C. C. Carbonell, Manuela B. Pucca

**Affiliations:** ^1^Medical School, Federal University of Roraima, Boa Vista, Roraima, Brazil; ^2^Post Graduate Program in Tropical Medicine (PPGMT) of the State University of Amazonas, Manaus, Amazonas, Brazil; ^3^Department of Teaching and Research, Dr. Heitor Vieira Dourado Tropical Medicine Foundation, Manaus, Amazonas, Brazil; ^4^Department of BioMolecular Sciences, School of Pharmaceutical Sciences of Ribeirão Preto, University of São Paulo, Ribeirão Preto, São Paulo, Brazil; ^5^Department of Biotechnology and Biomedicine, Technical University of Denmark, Kongens Lyngby, Denmark; ^6^Programa Doutoral de Bioética da Faculdade de Medicina do Porto, Cidade do Porto, Portugal; ^7^Department of Clinical Analysis, School of Pharmaceutical Sciences, São Paulo State University, Araraquara, São Paulo, Brazil

**Keywords:** snakebite envenoming (SBE), *Bothrops*, Roraima, ischemia, Yanomami, Amazon

## Abstract

Snakebites have a great impact in the Brazilian Amazon, being the lancehead *Bothrops atrox* the species responsible for most accidents, disabilities, and deaths. This study shows a case report of an indigenous patient from the Yanomami ethnicity, male, 33 years-old, envenomed by a *B. atrox* snake. Envenoming caused by *B. atrox* are characterized by local manifestations (e.g., pain and edema) and systemic manifestations, mainly coagulation disorders. The indigenous victim was admitted in the main hospital of Roraima and evolved with an unusual complication, an ischemia and necrosis of the proximal ileum, requiring segmental enterectomy with posterior side-to-side anastomosis. The victim was discharge after 27 days of hospitalization with no complaints. Snakebite envenomations may evolve with life-threatening complications, which can be treated by the antivenom following access to a healthcare unit, often late in indigenous population. This clinical case shows the need of strategies that aim improvement in the access to the healthcare by indigenous people, as well as demonstrates an unusual complication that may result from lancehead snakebites. The article also discusses the decentralization of snakebites clinical management to indigenous community healthcare centers to mitigate complications.

## 1. Introduction

Snakebite envenomations (SBEs) are considered an important public health problem (1). According to WHO, it is estimated that up to 2.7 million SBEs globally, resulting in approximately 400,000 disabilities, and 80,000 to 140,000 deaths per year (2).

According to the official SBEs' reporting system (*Sistema de Informações de Agravos de Notificação*, SINAN), Brazil reports ~30,000 snakebites per year (3), making this neglected disease an important public health problem. In this country, Roraima state stands out, presenting the highest SBEs' incidence rate (4), being the lancehead snake *Bothrops atrox* responsible for ~70% of the cases, followed by rattlesnakes' bites (3).

As other lancehead venoms, *B. atrox* venom cocktail presents proteolytic, coagulant, and hemorrhagic activities (5), resulting in hemostatic disturbances and tissue damage (6). Hemostatic disturbances can induce coagulopathies clinically manifested as hemorrhages and ecchymosis (1, 6). Bleedings may occur mostly by metalloprotease-induced activity (7, 8), promoting disturbances in the coagulation through factor X, II, and II activation, or indirectly, by factor VII activation (9). Although local bleeding is the most common, the venom may induce systemic bleeding in vital organs, such as brain, respiratory, and in the gastrointestinal tract. Although not frequent, liver hematoma and hypovolemic shock can also occur (9). Moreover, sepsis and disseminated intravascular coagulation (DIC) have also been reported (1). However, all systemic effects are factors associated to worse prognosis and can be life-threatening (9). According to the signs and symptoms, SBE severity can be classified as mild, moderate, or severe, which will define the antivenom dosage (5). Here, it will be discussed a lancehead snakebite case report of a Yanomami indigenous patient (Roraima, Brazil), 33 years-old, who evolved to mesenteric ischemia, a clinical outcome never documented for *B. atrox* envenomings.

Cases of mesenteric ischemia from SBEs are poorly documented. Rosenthal et al. (10) for the first time report a case of intestinal ischemia after snakebite caused by *Lachesis muta* in Costa Rica. In that case, the 64-year-old patient was envenomed, and after 3 days of hospital discharged, the victim had to return due to the abdominal pain, which were caused by an obstruction of the superior mesenteric artery, which led to necrosis of the ileum and of the cecum (10). Later, Barbey et al. (11) presented a case of a man bitten by a *B. lanceolatus*, which sought medical assistance 2 days after the bite. Although the patient received antivenom treatment, he died, and complete autopsy demonstrated multifocal thrombotic microangiopathy, resulting in endothelial, cerebral, interlobular renal, myocardial, pulmonary, and mesenteric damages, which caused cerebral, myocardial, and mesenteric infarctions (11). The presentation of only two cases reporting mesenteric ischemia from SBEs shows how uncommon and/or underreported this complication is.

## 2. Case report

The study was conducted in accordance with the Declaration of Helsinki, and the protocol was approved by the Research Ethic Committee of the Federal University of Roraima, under protocol number CAAE 70659917.3.0000.5302. Written informed consent was obtained from the patient to published all the included information.

A Yanomami indigenous man, 33 years old, inhabitant of the Waiamu community (Hakoma pole), in the state of Roraima, Brazilian Amazon, was bitten by a snake on the lateral malleolar region of the right foot. The bite occurred during the victim's hunting activity, at 11:00 am, on March 26, 2022 (day 0). The patient referred to identify the snake as a lancehead, popularly known in Brazil as *jararaca*, and uniquely represented in Roraima by the *B. atrox* species.

On the next day following the snakebite (day 1), the victim sought medical assistance at Surucucu base pole from the *Distrito de Saúde Especial Ind*í*gena Yanomami* (DSEI Yanomami), located in Alto Alegre city, reporting ankle pain, edema on the right malleolar region, and ecchymosis on the ipsilateral popliteal area. Moreover, the patient presented abdominal distension and intense pain, predominantly in the right iliac fossa. On physical examination, the patient was in a regular condition, eupneic, peripheral oxygen saturation of 96%, anicteric, acyanotic, afebrile, blood pressure of 110/70 mmHg, and heart rate of 94 bpm. The patient did not refer any comorbidities.

The patient received intravenous hydrocortisone and intramuscular promethazine, to avoid possible hypersensitivity reactions caused by heterologous antivenom. The snakebite was classified as severe; however, the patient received intravenously only 5 vials of the *Bothrops* antivenom (SAB), due to the lack of the total number of vials (*n* = 12) recommended by the Brazilian Minister of Health in severe cases.

On day 3 (March 29, 2022), the patient was transferred to *Hospital Geral de Roraima Rubens de Souza Bento* (HGR), the main hospital, placed in Boa Vista, the Roraima's capital. The patient was admitted to the major trauma department of the hospital, presenting abdominal pain, abdominal distension, edema, and ecchymosis in the popliteal region. The patient received intravenously metamizole, tenoxicam, and the intramuscular anti-tetanus vaccine. Another 12 vials of SAB were prescribed after the administration of intravenous hydrocortisone and intramuscular promethazine to avoid hypersensitivity reactions. A bladder catheterization showed hematuria. On day 4 (March 30, 2022), the patient evolved with worsening pain disseminated to the right flank, without irradiating to the back, but with signs of peritonitis (evidencing an acute abdomen). For analgesia, metamizole and ibuprofen were prescribed, and antibiotic therapy initiated with oral amoxicillin. The laboratorial tests indicate alterations of tested analytes (Table 1).

**Table 1:** Laboratory analysis of the patient's blood throughout the hospitalization days.

**Analyte**	**March**						**April**					
	**29**	**31**	**02**	**03**	**05**	**06**	**07**	**10**	**13**	**17**	**20**	**24**
Hb	16.10	**10.70**	**9.6**	**11.90**	**9.0**	**8.60**	**8.70**	**9.50**	**9.90**	**10.60**	**11.80**	**10.40**
Ht	45.30	**30.90**	**28**	**35**	**25.40**	**24.60**	**24.40**	**29.40**	**31.30**	**32.50**	**35.50**	**33.10**
Leucocytes	**15.84**	**12.41**	**10.32**	**14.18**	**14.67**	**13.40**	**13.55**	**11.66**	8.77	**12.64**	8.09	9.61
Neutrophils	**93**	**88.30**	**80**	**86.60**	**79.50**	**81.10**	**80.50**	**75.50**	65.30	**76.40**	59.70	49.40
Platelets	112	110	289	275	**556**	**641**	**715.00**	**739.00**	**740**	**538**	**437**	356
**Ca** ^2^^**+**^	**-**	**7.09**	**-**	**6.92**	**-**	**-**	**-**	**-**	**-**	**-**	**-**	**-**
Glucose	86.35	86.69	-	**48.09**	74.44	-	63.10	-	-	80.82	76.30	72.28
Urea	**59.30**	**53.05**	30.16	38.29	19.67	-	**10.82**	**8.31**	-	18.92	26.51	26.02
Creatinine	0.82	0.79	-	-	**0.64**	-	**0.56**	**0.59**	-	-	-	0.93
ALT	26.74	**50.99**	**84.28**	-	32.94	-	30.38	43.30	-	**50.62**	**49.66**	26.58
AST	**57.01**	**97.71**	42.55	**59.42**	**72.26**	-	43.92	38.90	-	**51.38**	38.16	18.20
Alkaline phosphatase	-	**106.71**	83.75	34.87	90.92	-	-	-	-	-	**103.51**	99.12
γGT	-	**49.08**	39.02	**49.85**	**75.89**	-	-	-	-	-	42.61	35.98
B_T_	**2.02**	**1.48**	-	**1.23**	-	-	0.50	0.27	-	-	-	0.27
B_D_	**0.57**	**0.61**	-	**0.65**	-	-	0.28	0.13	-	-	-	0.15
B_I_	**1.45**	**0.87**	-	0.58	-	-	0.22	0.14	-	-	-	0.12
Amylase	-	**77.33**	-	**105.31**	-	-	-	-	-	-	-	-
Lipase	-	**78.34**	-	-	-	-	-	-	-	-	-	-
Albumin	-	-	-	**2.17**	**1.80**	-	-	**1.95**	-	**2.91**	**3.26**	**3.30**
Procalcitonin	-	-	-	**0.59**	**0.53**	-	**0.21**	-	-	-	**0.13**	-
PT	**17.6**	**16.30**	**14.5**	**15.9**	**14.2**	-	13.9	**15.7**	-	**15.8**	12.8	**14.1**
PTT	**39.9**	35.53	35.6	31.6	38.5	-	35.5	31	-	**49.5**	30.8	32
INR	**1.27**	**1.24**	1.04	1.14	1.01	-	0.99	1.13	-	1.13	0.91	1.01
LDH	-	**866.55**	**751.82**	**513.53**	**497.96**	-	-	-	-	-	-	217.54
CRP	**134.08**	**142.88**	**135.12**	**143.06**	**133.82**	**115.87**	**108.35**	**70.79**	-	**8.85**	4.45	5.21
CK-MB	**48.78**	**45.54**	-	**28.07**	-	-	-	-	-	-	-	-
CPK	**241.73**	76.18	-	27.96	**200.32**	-	-	-	-	-	-	-

Bold values are outside the reference values, according to the Laboratory Nucleus of the General Hospital of Roraima (HGR). Hb (hemoglobin): 13.5–18 g/dL; Ht (hematocrit): 40–50%; Leucocytes: 4–10 × 10^3^/μL; Neutrophils: 50–70%; Platelets: 150–400 × 10^3^/μL; Ca^2+^ (calcium): 8.8–11 mg/dL; Glucose: 60–99 mg/dL; Urea: 15–40 mg/dL; Creatinine: 0.7–1.4 mg/dL; ALT (alanine aminotransferase): 5–48 U/L; AST (aspartate aminotransferase): 5–48 U/L; Alkaline phosphatase: 27–100 mg/dL; γGT (γ-glutamyl transpeptidase): 12–45 U/L; B_T_ (total bilirubin): 0.1–1.2 mg/dL; B_D_ (direct bilirubin): 0–0.4 mg/dL; B_I_ (indirect bilirubin): 0.1–0.8 mg/dL; Amylase: 125–349 U/L; Lipase: 0–60 U/L; Albumin: 3.5–5.5 g/dL; Procalcitonin: 0.01–0.12 ng/mL; PT (prothrombin time): 10–14 s; PTT (partial thromboplastin time): 25–39 s; INR (international normalized ratio): 0.8–1.2; LDH (lactate dehydrogenase): 200–480 U/L; CRP (C-reactive protein): 0–8 mg/dL; CK-MB (creatine kinase MB isoform): 0–25 U/L; CPK (creatine phosphokinase): 24–195 U/L.

**Figure 1:**
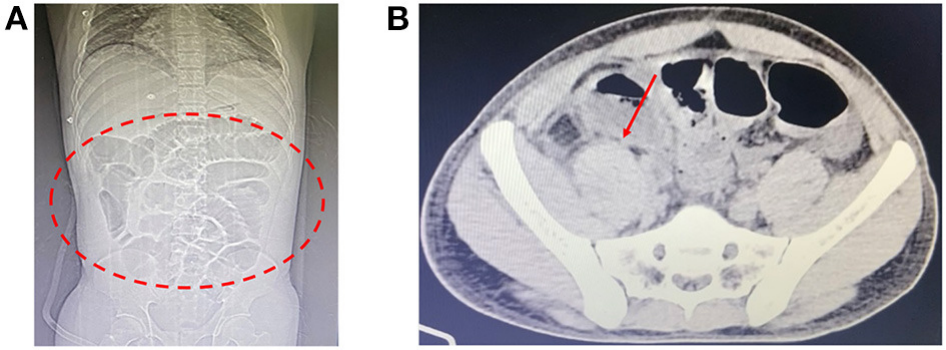
Computed tomography scans. **(A)** Coronal view showing dilated intestinal loops (red dashed circle). **(B)** Axial view indicating the presence of intraluminal fluid (red arrow).

On day 5 (March 31, 2022), a computed tomography (CT) scan of the abdomen and pelvis detected the presence of small ascites along with moderate distention of intestinal loops. The scan showed fluid accumulation and the presence of air-fluid level (Figure 1).

On day 6 (April 1, 2022) and 7 (April 2, 2022), the patient was kept under intravenous hydration, and medications for pain, fever, and nausea/emesis were maintained. Penicillin was also administered.

On day 8 (April 3, 2022), the patient underwent another CT of the abdomen, which again revealed a moderate amount of fluid in the abdominal cavity, in addition to distention of the small intestinal loops and thickening of the walls. He was then taken to the surgery room and underwent an exploratory laparotomy. During the procedure, ischemia and necrosis of the proximal ileum were observed. A segmental enterectomy was performed with a later side-to-side anastomosis. Ascitic fluid was collected during surgery for microbiological analyzes, which resulted in negative cultures. On the same day, a regimen of ceftriaxone and metronidazole was initiated.

On the following day (April 4, 2022), the physical examination revealed that the patient was in good condition, hydrated, afebrile, with a flat abdomen, without visceromegaly, with a clean surgical wound. The abdominal drain in the left flank showed a small amount of serosanguineous fluid, pain only on deep palpation in the right iliac fossa and right flank. The intravenous hydration was maintained, and clindamycin was prescribed.

**Figure 2:**
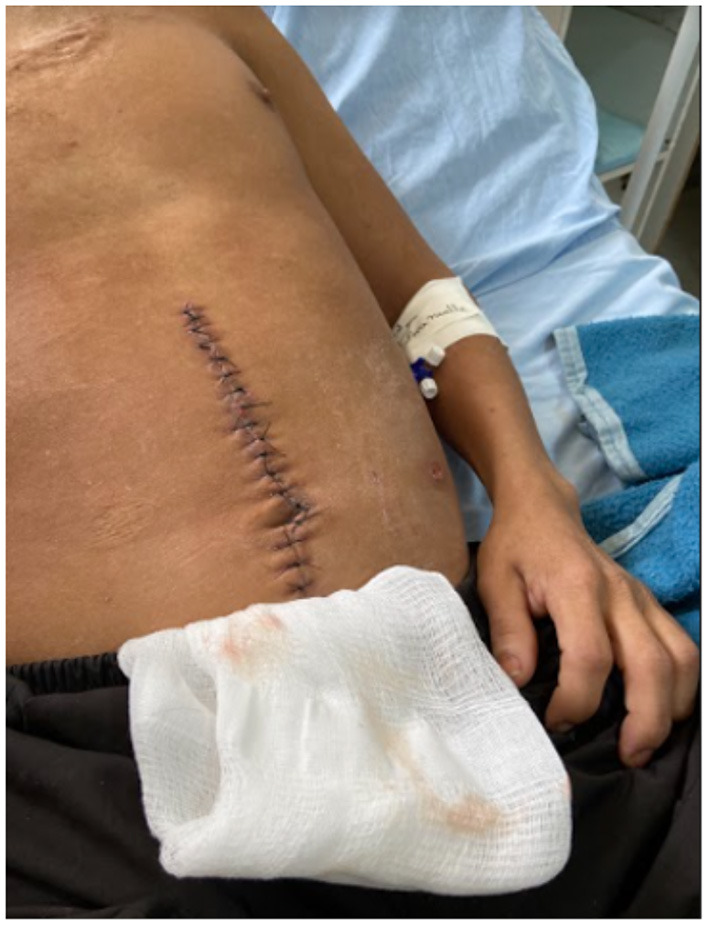
Surgical wound on the 10 postoperative day, demonstrating the healing progress at this stage.

On the 4th day after surgery (April 7, 2022) the patient remained afebrile in the last 24 h and with bowel movements present. On physical examination, the surgical wounds showed no signs of inflammation (Figure 2). The drain and indwelling urinary catheter were removed.

**Figure 3:**
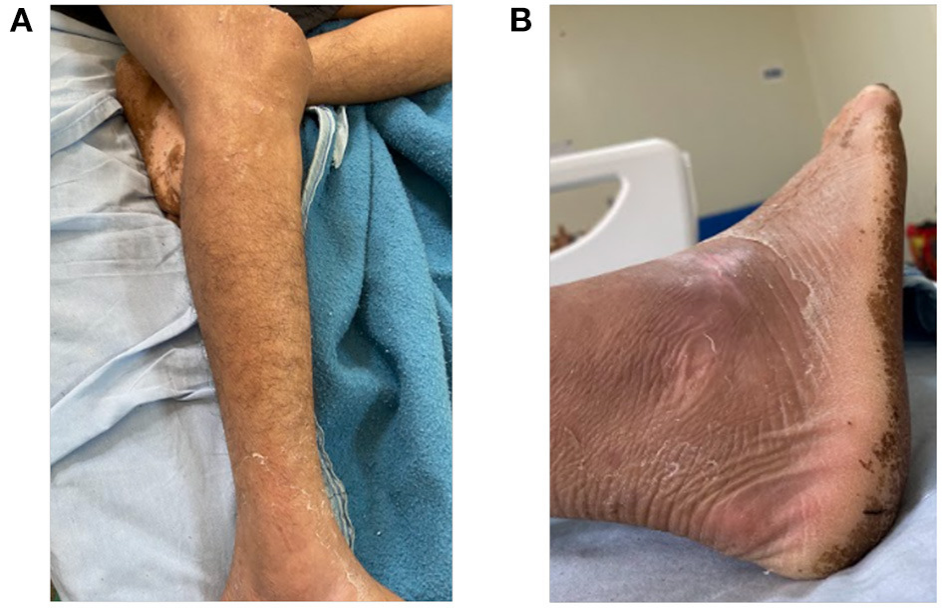
Status of the affected limb 17 days after the snakebite accident. **(A)** Leg and **(B)** foot.

On day 18 (April 13, 2022), the patient continued with clinical improvement, and with reduced edema of the affected lower limb (Figure 3). For a better assessment of the snakebite region, a soft tissue ultrasound of the right malleolar region was performed on April 14, 2022, which revealed a slight laminar infiltrative edema of the peri-articular cellular tissue in the ankle and dorsum of the foot, without other injuries. Ferrous sulfate and folic acid were also prescribed to the patient to reverse anemia (hemoglobin of 9.90 g/dL and hematocrit of 31.3%).

On day 22 (April 17, 2022), purulent secretion was observed in the surgical wound, which led the change the antibiotic therapy to ceftazidime and vancomycin. The new therapy was maintained for 7 days (till April 24, 2022), where the purulent secretion was no longer noticed. The patient was kept under observation for two more days (April 25–26, 2022).

In order to monitor the evolution of the condition, an ultrasound of abdomen was performed on April 20, 2022 (day 25), which showed a small discontinuity of the cutaneous plane in the topography of the epigastrium, and in the right iliac fossa, in addition to a small amount of infiltrated fluid in a laminar format in the subcutaneous cellular tissue without other larger free or encapsulated collections.

**Figure 4:**
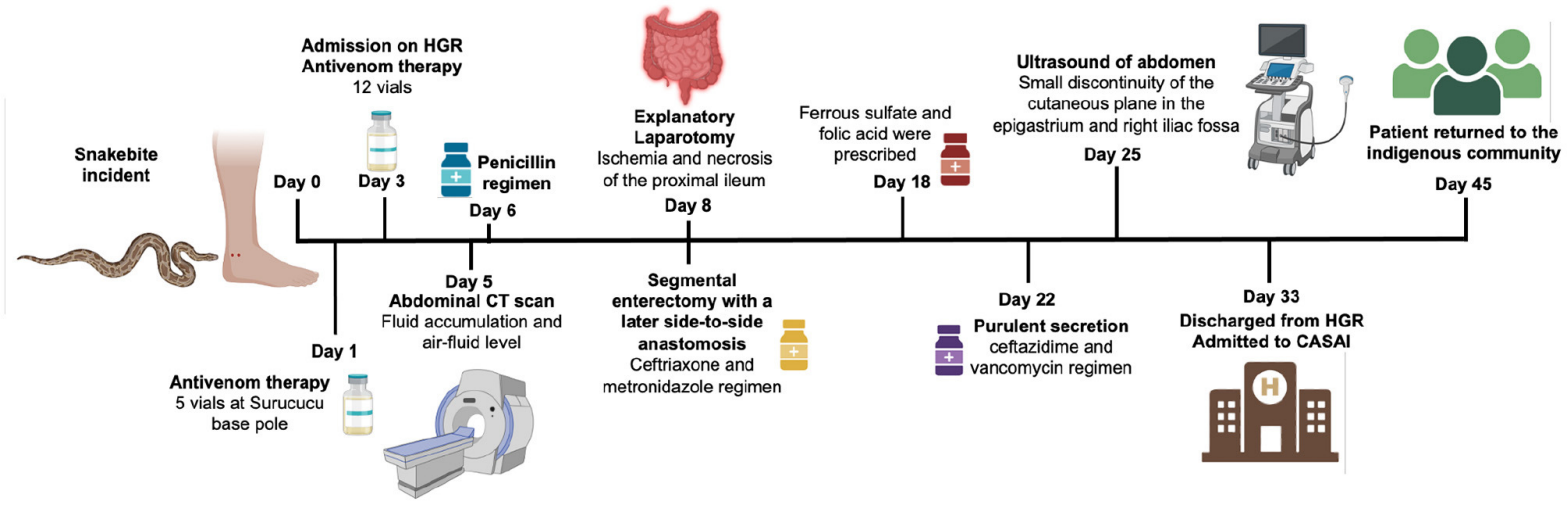
Timeline of the snakebite unusual case, illustrating the chronological sequence of events. Figure created with BioRender.com.

The patient presented a satisfactory clinical evolution during the hospitalization period. He was discharged from HGR hospital to *Casa do Índio* (CASAI), located in Boa Vista, Roraima, after 33 days of the snakebite (April 27, 2022) with a medical prescription of albendazole and ferrous sulfate. The patient stayed in CASAI a total of 13 days to complete the healing of the surgical wound. The patient returned to the community on May 10, 2022, 45 days after the accident (Figure 4).

## 3. Discussion

SBEs are considered a public health problem due to the high morbidity, and mortality, and the considerable socioeconomic impact (12). Rural, and indigenous communities in impoverished tropical and subtropical areas of the world are the most affected regions, such as Latin America (13, 14). In Brazil, there are ~30,000 SBEs per year, although it may be even higher due to underreporting and the difficulty to access health facilities (3, 15). Venomous snakes of public health interest in Brazil are divided into two families, Elapidae (*Micrurus*) and Viperidae (*Bothrops, Bothrocophias, Crotalus*, and *Lachesis*), being the most cases of SBEs caused by *Bothrops* genus popularly known as *jararacas* (15, 16). In Roraima, *B. atrox* is the only responsible for the recorded accidents in this genus, mainly due to its high degree of adaptability to different types of environments (pasture, crops, and even urban areas) (17).

The clinical case reported here converges with the epidemiological profile of SBEs in Brazil, in which most victims are male, of working age, and between 16 and 50 years old. The main place of occurrence of SBEs is rural areas, especially in indigenous lands, and the lower limbs are the most affected body region (85–90% of cases) (18, 19). In most cases, bite occurs during occupational activities, such as the hunting activity performed by the victim presented in this report (20).

The clinical classification of the SBE presented here is a severe *Bothrops* envenomation (18). It is important to emphasize that most severe cases occur because of the delay in medical care, and affect mainly the population of remote areas, such as indigenous peoples (12, 21). The Yanomami victim of this study sought medical attention 1 day after the bite, but only received the full antivenom dosage 3 days after the bite. This delay may have contributed negatively to the patient's prognosis.

The local manifestations presented by the patient in the present study are quite typical of what is reported in the literature for lanceheads (13, 22), that is, pain and edema, which progressively increased over the first days and affected a large part of the right lower limb. In addition to the local manifestations, the patient presented systemic bleeding, *i.e*., ecchymosis in the popliteal region, and hematuria. Among the hemorrhagic manifestations, bleeding from venipuncture, ecchymosis, bleeding at other sites of previous trauma or healed wounds, gingival bleeding, hemoptysis, macrohematuria, hematemesis and, in severe and lethal cases, hemorrhagic strokes may occur (23, 24). Indeed, the hemorrhagic syndrome caused by snakes of the *Bothrops* genus is the main cause of lethality (25, 26). Blood becomes incoagulable due to consumption coagulopathy usually within 1 h (27). Thrombocytopenia is an atypical event in envenomation in the Amazon region, and it is mainly caused by *B. atrox*, but is associated to systemic bleeding (26, 28, 29). The patient in this study arrived at the medical service with thrombocytopenia and mild unclottable blood, clinically manifested by hematuria and ecchymosis. These manifestations are caused mainly by metalloproteases (SVMPs), phospholipases A_2_, and serine proteases (SVSP), occurring in 6–8 h after the snakebite (30). On the other hand, some hemorrhagic manifestations (e.g., central nervous system bleeding) may occur even with normal platelet counts and clotted blood (24, 31).

The patient of the study also developed significant abdominal pain in the region of the right flank and right iliac fossa, arising from an ischemic manifestation evidenced in an abdominal CT, mesenteric ischemia, necrosis of the proximal ileum with distension, and thickening of the small intestinal loops. Ischemic complications of *Bothrops* snakebites are unusual and the responsible mechanisms are not fully understood (32, 33). There are very few cases reporting ischemic strokes such as a 6-year-old child admitted 4 days after the snakebite (32) and a 50-year-old man who despite early care evolved with the complication (34). Among the procoagulant toxins of *Bothrops* venom, there are enzymes with thrombin-like activity classified as SVSP, that directly hydrolyze fibrinogen into fibrin, especially Batroxobin, in addition to BA III-4 SVSP and Thrombocytin, which, in addition to thrombin-like activity, promotes activation of platelet aggregation and coagulation factors XIII and VIII (6, 33, 35).

Furthermore, disturbances in hemostasis are also caused by toxins like SVMPs, which promote the direct activation of factors II and X, resulting in the formation of endogenous thrombin (36). The activation of these factors can lead to the formation of microthrombi that can cause distant visceral infarctions, such as ischemic stroke and soft tissue ischemia and necrosis (32). Thrombotic complications usually occur within 5 h to 1 week after the snakebite and are multifocal, occlusive microthrombi can affect cerebral, myocardial, pulmonary, femoral and mesenteric arteries (33).

Venom composition among *Bothrops* species is variable, and most of the intraspecific variability of *B. atrox* venom is related to components involved in hemostatic disorders (6). Fortunately, despite differences in toxicity, procoagulant activity is neutralized by antivenoms (37). Hemostatic complications are essentially preventable with the only form of specific treatment currently existing for snakebites, antivenoms, but the success of this therapy requires its adequate administration, with a quantity of ampoules compatible with the severity of the condition and preferably within the first 6 h after the accident (6, 38). However, vulnerable populations can take days to reach health services, and the serum can be no longer able to neutralize the effects of the envenomation (6). In the case of the patient in this report, neither the ideal administration time of the antivenom nor the number of ampoules were initially respected, negatively impacting his clinical evolution. Despite it, the patient made an excellent recovery after the surgical treatment and returned to his community after the unusual envenomation.

For instance, to produce botropic antivenom, horses are hyperimmunized with venom from snakes such as *B. alternatus, B. jararaca, B. jararacussu, B. moojeni*, and *B. neuwiedi* (39). However, it is important to note that the venom pool used in production may or may not contain all the components present in the venom of *B. atrox* (40, 41). Furthermore, there can be variations in venom components even within the same species or subspecies, making it more challenging to neutralize all venom components effectively (42). Consequently, the venom of *B. atrox* may contain different components in terms of quality and quantity, which can influence the severity of envenomation cases. Ultimately, the severity of an envenomation case depends on various factors, including the species involved, the quantity and potency of the venom injected, the time elapsed before treatment, the individual's immune response, and the availability and administration of appropriate medical care (43).

It is important to point out that the victim in this case was out of his community for 45 days and despite not having anatomical, and/or functional sequelae, he had psychological suffering due to the distance from his community, and culture, in addition of having incurred expenses for the public healthcare system.

## 4. Conclusion

In conclusion, SBEs demand complex medical care that goes far beyond the administration of antivenom. Moreover, snakebite outcomes can vary a lot, resulting even in mesenteric ischemia as reported here. Based on that, clinicians should be aware when the snakebite victim reports abdominal pain, requesting quickly imaging tests and, if necessary, promptly perform a surgical procedure, since unexpected outcomes require rapid medical action to save the victim's life.

## Data availability statement

The original contributions presented in the study are included in the article/supplementary material, further inquiries can be directed to the corresponding author.

## Ethics statement

The studies involving human participants were reviewed and approved by the Research Ethic Committee of the Federal University of Roraima, under protocol number CAAE 70659917.3.0000.5302. The patients/participants provided their written informed consent to participate in this study. Written informed consent was obtained from the patient to publish all the included information.

## Author contributions

LG, VSoS, VSaS, DD, and RC followed the patient and managed the snakebite. RM, SJ, IO, FC, WM, and JS contributed to the drafting, discussion, and revision of the article. MP is the coordinator of the Snakebite Roraima research group, supervised the students, and design the study. All authors contributed to the article and approved the submitted version.

## References

[1] Oliveira IS Ananias CB Medeiros JM Franco MVS Ferreira IG Cerni FA. Medical management after lancehead snakebite in north amazon: a case report of long-term disability. Toxins. (2022) 14:494. 10.3390/toxins14070494 35878232 PMC9319475

[2] World Health Organization. Snakebite Envenoming: A Strategy for Prevention and Control. Geneva: World Health Organization (2019). Available online at: https://apps.who.int/iris/handle/10665/324838 (accessed September 5, 2022).

[3] Ministério da Saúde,. Acidentes Por Animais Peçonhentos - Notificações Registradas no Sistema de Informação de Agravos de Notificação - Brasil. (2022). Available online at: http://tabnet.datasus.gov.br/cgi/deftohtm.exe?sinannet/cnv/animaisbr.def (accessed April 26, 2022).

[4] Melo E,. C. (2018). Perfil epidemiológico dos acidentes ofí*dicos no Estado de Roraima de 2013 a 2016* (Thesis). Universidade federal de Roraima, Boa Vista, Brazil. Available online at: https://webcache.googleusercontent.com/search?q=cache:vq0QQ60qCykJ:https://ufrr.br/procisa/index.php%3Foption%3Dcom_phocadownload%26view%3Dcategory%26download%3D897:perfil-epidemiologico-dos-acidentes-ofidicos-no-estado-de%26id%3D63:dissertacoes-2018%26Itemid%3D277&cd=2&hl=pt-BR&ct=clnk&gl=br">https://ufrr.br/procisa/index.php%3Foption%3Dcom_phocadownload%26view%3Dcategory%26download%3D897:perfil-epidemiologico-dos-acidentes-ofidicos-no-estado-de%26id%3D63:dissertacoes-2018%26Itemid%3D277&cd=2&hl=pt-BR&ct=clnk&gl=br (accessed September 5, 2022).

[5] Tavares-Neto J. Manual de diagnóstico e tratamento de acidentes por animais peçonhentos. Rev Baiana de Saúde Pública. (2014) 76:156. 10.22278/2318-2660.1996.v1.n2.a1256

[6] Monteiro WM Contreras-Bernal JC Bisneto PF Sachett J Mendonça da Silva I Lacerda M. Bothrops atrox, the most important snake involved in human envenomings in the amazon: How venomics contributes to the knowledge of snake biology and clinical toxinology. Toxicon: X. (2020) 6:100037. 10.1016/j.toxcx.2020.100037 32550592 PMC7285970

[7] Escalante T Rucavado A Fox JW Gutiérrez JM. Key events in microvascular damage induced by snake venom hemorrhagic metalloproteinases. J Proteomics. (2011) 74:1781–94. 10.1016/j.jprot.2011.03.026 21447411

[8] Yamashita KM Alves AF Barbaro KC Santoro ML. Bothrops jararaca venom metalloproteinases are essential for coagulopathy and increase plasma tissue factor levels during envenomation. PLoS Negl Trop Dis. (2014) 8:e2814. 10.1371/journal.pntd.0002814 24831016 PMC4022520

[9] Cunha FC Heerdt M Torrez PPQ França FO de S Molin GZD Battisti R. First report of hepatic hematoma after presumed *Bothrops* envenomation. Rev Soc Bras Med Trop. (2015) 48:633–5. 10.1590/0037-8682-0084-2015 26516980

[10] Rosenthal R Meier J Koelz A Müller C Wegmann W Vogelbach P. Intestinal ischemia after bushmaster (*Lachesis muta*) snakebite—a case report. Toxicon. (2002) 40:217–20. 10.1016/S0041-0101(01)00203-3 11689244

[11] Barbey C Malbranque S Smadja D Courcier D Warrell DA Piercecchi-Marti MD. Fatal diffuse thrombotic Microangiopathy after a Bite by the “Fer-de-Lance” Pit Viper (*Bothrops lanceolatus*) of Martinique. Am J Trop Med Hyg. (2008) 78:856–61. 10.4269/ajtmh.2008.78.856 18541759

[12] Hui Wen F Monteiro WM Moura da Silva AM Tambourgi DV Mendonça da Silva I Sampaio VS. Snakebites and scorpion stings in the Brazilian Amazon: identifying research priorities for a largely neglected problem. PLoS Negl Trop Dis. (2015) 9:e0003701. 10.1371/journal.pntd.0003701 25996940 PMC4440781

[13] Gutiérrez JM Calvete JJ Habib AG Harrison RA Williams DJ Warrell DA. Snakebite envenoming. Nat Rev Dis Primers. (2017) 3:17063. 10.1038/nrdp.2017.63 28905944

[14] Harrison RA Hargreaves A Wagstaff SC Faragher B Lalloo DG. Snake envenoming: a disease of poverty. PLoS Negl Trop Dis. (2009) 3:e569. 10.1371/journal.pntd.0000569 20027216 PMC2791200

[15] Lemos J de C Almeida TD de Fook SML Paiva A de A Simões MO da S. Epidemiologia dos acidentes ofídicos notificados pelo Centro de Assistência e Informação Toxicológica de Campina Grande (Ceatox-CG), Paraíba. Revista Brasileira de Epidemiol. (2009) 12:50–9. 10.1590/S1415-790X2009000100006

[16] Matos RR Ignotti E. [Incidence of venomous snakebite accidents by snake species in Brazilian biomes]. Cien Saude Colet. (2020) 25:2837–46. 10.1590/1413-81232020257.31462018 32667565

[17] Oliveira M Martins M. When and where to find a pitviper: activity patterns and habitat use of the lancehead, Bothrops atrox, in Central Amazonia, Brazil. Herpetological Natural History. (2001) 8:101–9.

[18] Príncipe Azevedo LR da Cruz Rodrigues K Rodrigues Macedo VP Arruda de. Faria C. Perfil clínico-epidemiológico dos acidentes ofídicos ocorridos no. Brasil SaudColetiv. (2021) 11:4876–87. 10.36489/saudecoletiva.2021v11i61p4876-4887

[19] Feitosa ES Sampaio V Sachett J Castro DB de Noronha M. das DN. Snakebites as a largely neglected problem in the Brazilian Amazon: highlights of the epidemiological trends in the State of Amazonas. Revista da Sociedade Brasileira de Medicina Tropical. (2015) 48:34–41. 10.1590/0037-8682-0105-2013 26061369

[20] Magalhães SFV Peixoto HM Moura N Monteiro WM de Oliveira MRF. Snakebite envenomation in the Brazilian Amazon: a descriptive study. Trans R Soc Trop Med Hyg. (2019) 113:143–51. 10.1093/trstmh/try121 30476298

[21] Schneider MC Vuckovic M Montebello L Sarpy C Huang Q Galan DI. Snakebites in rural areas of Brazil by race: indigenous the most exposed group. Int J Environ Res Public Health. (2021) 18:365. 10.3390/ijerph18179365 34501955 PMC8431164

[22] Ministério da Saúde, Fundação Nacional de, Saúde,. Manual de Diagnóstico e Tratamento de Acidentes por Animais Peçonhentos. (2001). Available online at: https://www.icict.fiocruz.br/sites/www.icict.fiocruz.br/files/Manual-de-Diagnostico-e-Tratamento-de-Acidentes-por-Animais-Pe–onhentos.pdf

[23] Pardal PP de O Souza SM Monteiro MR de C da C Fan HW Cardoso JLC França FOS. Clinical trial of two antivenoms for the treatment of Bothrops and Lachesis bites in the north eastern Amazon region of Brazil transactions of the royal society of tropical. Med Hygiene. (2004) 98:28–42. 10.1016/S0035-9203(03)00005-1 14702836

[24] Pérez-Gómez AS Monteiro WM João GAP Sousa JD de B Safe IP Damian MM. Hemorrhagic stroke following viper bites and delayed antivenom administration: three case reports from the Western Brazilian Amazon. Revista da Sociedade Brasileira de Medicina Tropical. (2019) 52:2019. 10.1590/0037-8682-0115-2019 31340373

[25] da Silva Souza A de Almeida Gonçalves Sachett J Alcântara JA Freire M Alecrim M. Snakebites as cause of deaths in the Western Brazilian Amazon: why and who dies? Deaths from snakebites in the Amazon. Toxicon. (2018) 145:15–24. 10.1016/j.toxicon.2018.02.041 29490236

[26] Silva de Oliveira S Campos Alves E dos Santos Santos A Freitas Nascimento E Tavares Pereira JP Mendonça da Silva I. Bothrops snakebites in the Amazon: recovery from hemostatic disorders after Brazilian antivenom therapy. Null. (2020) 58:266–74. 10.1080/15563650.2019.1634273 31264481

[27] Otero-Patiño R. Epidemiological, clinical and therapeutic aspects of Bothrops asper bites. Toxicon. (2009) 54:998–1011. 10.1016/j.toxicon.2009.07.001 19591857

[28] Oliveira S Alves E. Santos A. Nascimento E. Pereira J. P. Silva M.. (2020). Bleeding disorders in bothrops atrox envenomations in the brazilian amazon: Participation of hemostatic factors and the impact of tissue factor. Toxins. 12, 554. 10.3390/toxins12090554 32872404 PMC7551922

[29] Oliveira SS Alves EC Santos AS Pereira JPT Sarraff LKS Nascimento EF. Factors associated with systemic bleeding in bothrops envenomation in a tertiary hospital in the Brazilian amazon. Toxins. (2019) 11:22. 10.3390/toxins11010022 30621001 PMC6356762

[30] Sajevic T Leonardi A KriŽaj I. Haemostatically active proteins in snake venoms. Toxicon. (2011) 57:627–45. 10.1016/j.toxicon.2011.01.006 21277886

[31] de Oliveira SS Freitas-de-Sousa LA Alves EC de Lima Ferreira LC da Silva IM de Lacerda MVG. Fatal stroke after Bothrops snakebite in the Amazonas state, Brazil: A case report. Toxicon. (2017) 138:102–6. 10.1016/j.toxicon.2017.08.021 28842354

[32] Assamadi M. M Barek YA, Elallouchi Y, Benantar L, Aniba K. [Ischemic stroke, an unusual complication of snake bite: case report]. Pan Afr Med J. (2022) 41:50. 10.11604/pamj.2022.41.50.22225 35317478 PMC8917463

[33] Larréché S Chippaux J-P Chevillard L Mathé S Résière D Siguret V. Bleeding and thrombosis: insights into pathophysiology of bothrops venom-related hemostasis disorders. International J Mol Sci. (2021) 22:643. 10.3390/ijms22179643 34502548 PMC8431793

[34] Martínez-Villota VA Mera-Martínez PF Portillo-Miño JD. Massive acute ischemic stroke after Bothrops spp. envenomation in southwestern Colombia: Case report and literature review. Biomedica. (2022) 42:9–17. 10.7705/biomedica.6114 35471166 PMC9045098

[35] Niewiarowski S Kirby EP Brudzynski TM Stocker K. Thrombocytin, a serine protease from Bothrops atrox venom. 2 Interaction with platelets and plasma-clotting factors. Biochemistry. (1979) 18:3570–7. 10.1021/bi00583a021 476069

[36] Hofmann H Bon C. Blood coagulation induced by the venom of Bot hrops atrox. 1 Identification, purification, and properties of a prothrombin activator. Biochemistry. (1987) 26:772–80. 10.1021/bi00377a018 3552031

[37] Del-Rei THM Sousa LF Rocha MM Freitas-de-Sousa LA Travaglia-Cardoso SR Grego K. Functional variability of Bothrops atrox venoms from three distinct areas across the Brazilian Amazon and consequences for human envenomings. Toxicon. (2019) 164:61–70. 10.1016/j.toxicon.2019.04.001 30991062

[38] Larréché S Mion G Goyffon M. Troubles de l'hémostase induits par les venins de serpents. Annal Franç Réanimation. (2008) 27:302–9. 10.1016/j.annfar.2008.02.009 18420371

[39] Barreto GNLS Oliveira SS de Anjos IV Chalkidis H de M Mourão RHV Moura-da-Silva AM. Experimental Bothrops atrox envenomation: Efficacy of antivenom therapy and the combination of Bothrops antivenom with dexamethasone. PLOS Neglected Tropical Diseases. (2017) 11:e0005458. 10.1371/journal.pntd.0005458 28306718 PMC5371371

[40] Furtado M de FD Cardoso ST Soares OE Pereira AP Fernandes DS Tambourgi DV. Antigenic cross-reactivity and immunogenicity of Bothrops venoms from snakes of the Amazon region. Toxicon. (2010) 55:881–7. 10.1016/j.toxicon.2009.12.014 20036275

[41] Sousa LF Nicolau CA Peixoto PS Bernardoni JL Oliveira SS Portes-Junior JA. Comparison of phylogeny, venom composition and neutralization by antivenom in diverse species of bothrops complex. PLOS Neglected Trop Diseases. (2013) 7:e2442. 10.1371/journal.pntd.0002442 24069493 PMC3772048

[42] Chippaux J-P Williams V White J. Snake venom variability: methods of study, results and interpretation. Toxicon. (1991) 29:1279–303. 10.1016/0041-0101(91)90116-9 1814005

[43] Mise Y Lira-da-Silva R Carvalho F. Time to treatment and severity of snake envenoming in Brazil. Rev Panam Salud Publica. (2018) 5:1–6. 10.26633/RPSP.2018.52 31093080 PMC6386102

